# Efficient Second Harmonic Generation in 3D Nonlinear Optical-Lattice-Like Cladding Waveguide Splitters by Femtosecond Laser Inscription

**DOI:** 10.1038/srep22310

**Published:** 2016-02-29

**Authors:** Weijie Nie, Yuechen Jia, Javier R. Vázquez de Aldana, Feng Chen

**Affiliations:** 1 School of Physics, State Key Laboratory of Crystal Materials, and Key Laboratory of Particle Physics and Particle Irradiation (Ministry of Education), Shandong University, 250100 Jinan, Shandong, China; 2 Laboratory for Optical Systems, Department of Microsystems Engineering-IMTEK, University of Freiburg, 79110 Freiburg, Germany; 3 Laser Microprocessing Group, Universidad de Salamanca, 37008 Salamanca, Spain

## Abstract

Integrated photonic devices with beam splitting function are intriguing for a broad range of photonic applications. Through optical-lattice-like cladding waveguide structures fabricated by direct femtosecond laser writing, the light propagation can be engineered via the track-confined refractive index profiles, achieving tailored output beam distributions. In this work, we report on the fabrication of 3D laser-written optical-lattice-like structures in a nonlinear KTP crystal to implement 1 × 4 beam splitting. Second harmonic generation (SHG) of green light through these nonlinear waveguide beam splitter structures provides the capability for the compact visible laser emitting devices. With Type II phase matching of the fundamental wavelength (@ 1064 nm) to second harmonic waves (@ 532 nm), the frequency doubling has been achieved through this three-dimensional beam splitter. Under 1064-nm continuous-wave fundamental-wavelength pump beam, guided-wave SHG at 532 nm are measured with the maximum power of 0.65 mW and 0.48 mW for waveguide splitters (0.67 mW and 0.51 mW for corresponding straight channel waveguides), corresponding to a SH conversion efficiency of approximately ~14.3%/W and 13.9%/W (11.2%/W, 11.3%/W for corresponding straight channel waveguides), respectively. This work paves a way to fabricate compact integrated nonlinear photonic devices in a single chip with beam dividing functions.

Miniature nonlinear photonic devices, based on the optical waveguide technology, are desirable configurations owing to the combination of the nonlinear feature of bulks and on-demand geometries of waveguides for a broad range of photonic applications, including but not limited to optical telecommunications, sensing for atmosphere or biology, quantum computing and electro-optic modulators, etc^1^,^2^,^3^,^4^,^5^,^6^,^7^,^8^. As one of the most widely used nonlinear optical crystal, potassium titanyl phosphate (KTiOPO_4_ or KTP) is an ideal candidate due to its superior properties such as broad transmitting range, large nonlinear optical coefficients, high optical damage threshold, and broad thermal and angular acceptances for second harmonic generation^9^,^10^, which enables it one of the preferred nonlinear material for visible laser generation. In practice, the frequency doubling of the near infrared into the green, blue and yellow light has been successfully realized by using birefringent (KTP) or periodically poled potassium titanyl phosphate (PPKTP) wafers^11^,^12^,^13^,^14^. Optical waveguide micro-structure, as the basic component, can confine the light propagation in small volumes, reaching much higher optical density with respect to the bulks, which may considerably enhance some performances^15^ (such as the nonlinear properties^16^,^17^,^18^) relevant to the bulk materials in various waveguide configurations. Benefiting from the above mentioned advantages, second harmonic generation (SHG) in nonlinear waveguide structure can be realized under lower light powers, providing higher SH efficiency and more choice of different modes than the bulks^19^. The microstructure with function of beam splitting, with respect to the typical straight channel waveguides that strongly limit the actual control of the spatial properties for the laser^15^, have more advantages for the applications of monolithically integrated optical heterodyne systems and wavelength division multiplexing in the optical networks^20^. For the direct-pump waveguide laser, splitter waveguide shows inferior performance to the straight channel waveguide due to the laser cavity bending^21^. However, such drawback may be avoided in nonlinear waveguiding structures because the frequency conversion is less sensitive to the waveguide cavity with respect to the case of lasing. In addition, with certain designs, the 3D nonlinear waveguide splitters may possess not only low additional losses but also enhanced SH conversion efficiency. The KTP crystal is promising to achieve efficient combination of the waveguide splitter microstructure and excellent nonlinear bulk performance.

To fabricate the waveguides, various approaches could be utilized in a wide range of materials^22^,^23^. As one of the most efficient methods, femtosecond laser inscription has been widely applied to implement three dimensional one-step micro-processing in diverse transparent materials for a great variety of applications^25^,^26^,^27^,^28^,^29^,^30^,^31^,^32^ since the pioneering work by Davis *et al.* in 1996^24^. During the processing, the femtosecond laser beam is focused on a sample spot inducing extremely localized modifications of material matrix through nonlinear processes (i.e., two-photon or multiphoton absorption) followed by strong-field ionization^6^. Then the refractive index could be changed to be either positive or negative in the irradiated regions. For the positive refractive index changes (Δn > 0) such as induced in glasses^33^, it is easy to control the splitter geometries of the waveguide structures as a result of the guiding core located in the femtosecond laser modification regions. Whereas in the dielectric crystals, the femtosecond laser pulses usually induce negative changes (Δn < 0) of the refractive index^34^,^35^,^36^,^37^,^38^,^39^, which lead to certain intractability for 3D guiding implementation on account of the waveguide situated in the surrounding regions of the femtosecond-laser induced tracks. The hexagonal optical-lattice-like microstructure, in which a guiding core is surrounded by periodically arrayed laser-induced tracks with negative index modifications, has been realized for the first time in the Nd:YAG laser crystal^40^. By introducing the modification at arbitrary designed depths inside the substrates directly, on-demand construction of waveguides can be obtained for flexible light propagation, which promotes the development of innovative and unique device architectures. In this work, monolithic 3D optical-lattice-like cladding waveguide beam splitters have been experimentally implementation, in which unmodified cores are surrounded by hexagonal or dual-hexagonal lattices of tracks (Δn < 0) with induced defects capable of efficient light confinement in a wide spectral range. Femtosecond laser writing is applied for the fabrication of the splitter structure with designed geometries owing to its flexibility and conveniences, providing the possibility of simultaneous second harmonic generation (SHG) and tailoring of guided light beams. The SHG performance at ~532 nm has been realized in the structure regions under the pump of continuous wave (cw) fundamental waves at wavelength of ~1064 nm, which is compared between the waveguide splitters and corresponding straight channel waveguides.

## Results

**Figure 1:**
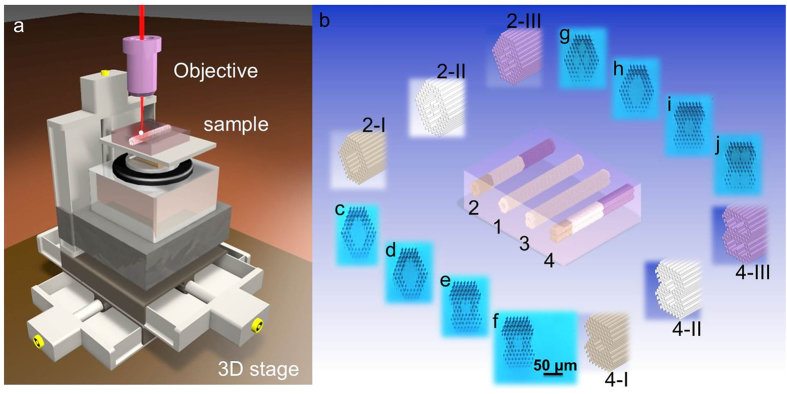
The fabrication of optical-lattice-like cladding waveguides. (**a**) The fabrication schematic plots of femtosecond laser-written optical-lattice-like cladding waveguides in KTP crystal. (**b**) The structures of optical-lattice-like cladding waveguides Nos. 1–4 in details. 2-I, II, III or 4-I, II, III is cross-sectional sketch of three part in waveguides Nos. 2 or 4., respectively. (**c**–**f**) The microscope images of input end face in optical-lattice-like cladding waveguides Nos 1–4, separately. (**g–j**) the microscope images of output end face in optical-lattice-like cladding waveguides Nos 1–4, separately.

The optical-lattice-like cladding waveguides in a KTP crystal wafer are fabricated by femtosecond-laser inscription technique. Figure 1(a) depicts the process of the femtosecond (fs) laser inscription. Focused fs-laser pulse produces localized modification in the focal volume forming the damage track, which depends on the parameters of the applied fs-laser pulse such as pulsed energy, repetition rate, pulse duration, scanning speed and beam polarization. Figure 1(b) demonstrates the schematic plot of the 3D optical-lattice-like cladding waveguides and its detailed cross-sectional topography. The inserts are the optical microscope images of the input (as shown in Fig. 1(c–f)) and output (as shown in Fig. 1(g–j)) facet in waveguides Nos. 1–4, respectively. The waveguide splitters consist of an unmodified core surrounded by hexagonally or double hexagonally arrayed track lattice, whose tracks have a transverse length of 10 μm, and the separation between two adjacent tracks is 8 μm. There are two different kinds of 1 to 4 waveguide splitters (i.e. waveguides No. 2 and No. 4) in the sample and two straight guiding structures (i.e. waveguides No. 1 and No. 3) are manufactured for comparison, correspondingly. The structures are produced by introducing specially designed axial defect lines in the core regions at different parts of the whole prototypes directly. To realize effective confinement of light beams, the three-dimension 1 to 4 waveguide splitters are fabricated by combining three designed elements with smoothly changing guiding cores, which are connected in sequence. In other words, the arbitrarily complex structures could be designed by introducing intermediated elements with on-demand axial track defects at certain positions, which is an intriguing and promising technique for various photonic applications. In waveguide No. 2, the lengths of three elements (i.e. 2-I, 2-II, 2-III) are 1.4 mm, 2.8 mm and 2.8 mm, respectively. While the 4-I, 4-II, and 4-III correspond to 1 mm, 3 mm and 3 mm in waveguide No. 4. In addition, waveguides No. 1 and No. 3 are same as the first element of waveguides No. 2 and No. 4 (i.e. 2-I and 4-I), correspondingly, whose unmodified cores are approximately a region with area of 50 × 30 μm^2^ and 30 × 30 μm^2^, respectively.

**Figure 2:**
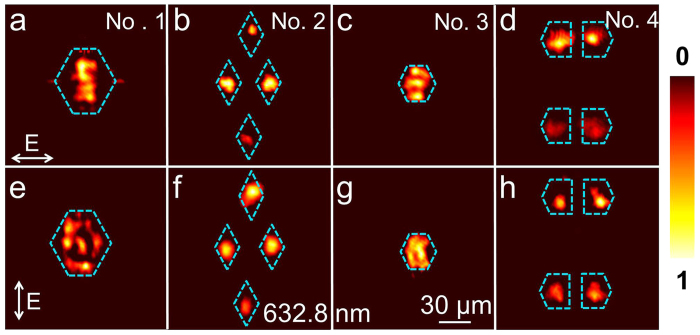
Guiding properties of optical-lattice-like cladding waveguides. Measured near-field intensity distribution of optical-lattice-like cladding waveguides Nos. 1–4 for (**a–d**) TE_00_ and (**e–h**) TM_00_ polarizations at 632.8 nm.

**Figure 3:**
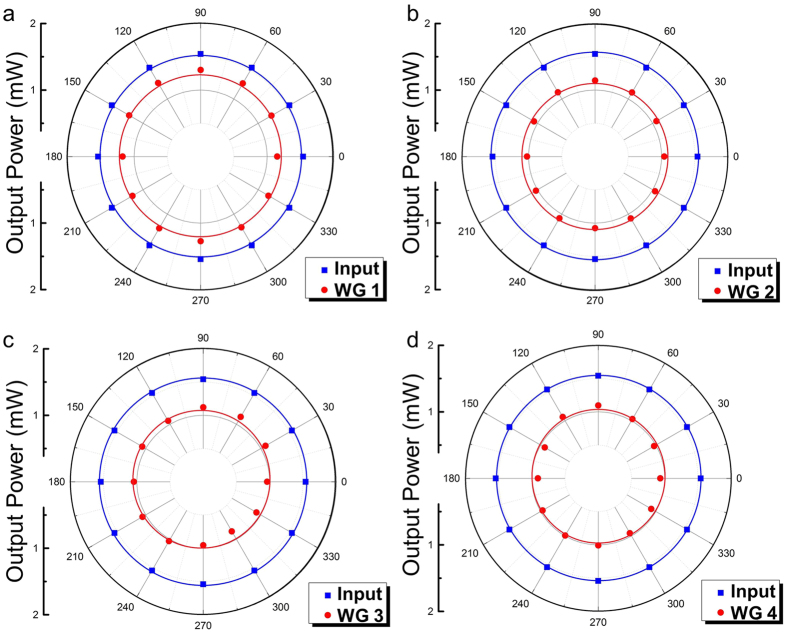
Polarization properties of optical-lattice-like cladding waveguides (**a**–**d**) All-angle light transmission along the transverse plane at 633 nm in optical-lattice-like cladding waveguides Nos 1–4, respectively.

To experimentally investigate the guiding properties of the 3D optical-lattice-like cladding waveguides, an end-face coupling arrangement at wavelength of 632.8 nm with a linearly-polarized continuous-wave (cw) laser is employed. Figure 2(a–h) illustrate the near-field intensity distribution of the waveguides Nos. 1–4 at transverse electric and transverse magnetic polarization, respectively. It could be found that the optical-lattice-like cladding waveguides implement two different ways of beam tailoring, which show excellent performance for 3D beam splitter function in the mutually perpendicular orientation. Considering the mismatch coefficient which can be calculated by an overlap integral between both the launched Gaussian field profiles and the modal profiles of the structure, the total attenuations (including splitting losses and guiding propagation losses) are determined to be 1.20 dB, 1.31 dB, 1.39 dB, 1.46 dB for optical-lattice-like cladding waveguides Nos. 1–4, respectively. The mismatch coefficient *C* can be expressed as^41^,^42^


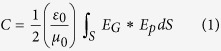


where the incident field *E*_*G*_ is assumed as the Gaussian field and the waveguide mode is *E*_*P.*_With an assumption of a 25-μm-diameter input Gaussian field *E*_*G*_ (estimated by experimental experience), the values which can be calculated by FD-BPM algorithm (Rsoft® Beam PROP)^42^ are ~0.72 and 0.88 for waveguides Nos. 1–4, respectively. By comparing the total attenuation of the beam splitter No. 2 (No. 4) and corresponding straight waveguides No. 1 (No. 3) with the same input end face, the additional losses of the three-dimension splitters are determined to be ~0.11 dB (0.07 dB). The splitting losses could be partly attributed to the imperfections of the structures. However, the obtained values are significantly smaller than those reported femtosecond laser written 1 × 3 splitters in pure fused silica^43^ (the splitting losses ~6 dB). In order to achieve thorough information of the polarization effects for propagating properties, the all-angle light transmission along the transverse plane is measured in waveguides Nos. 1–4 as shown in Fig. 3(a–d), respectively. With the same launched power at any polarization, the output power is nearly unaltered for the straight and splitter optical-lattice-like cladding waveguides (i.e. the guidance is almost polarization-independent). It is worth pointing out that the structures are capable of confining light transmission in any polarization, which is one of the necessary preconditions for birefringent phase matching.

**Figure 4:**
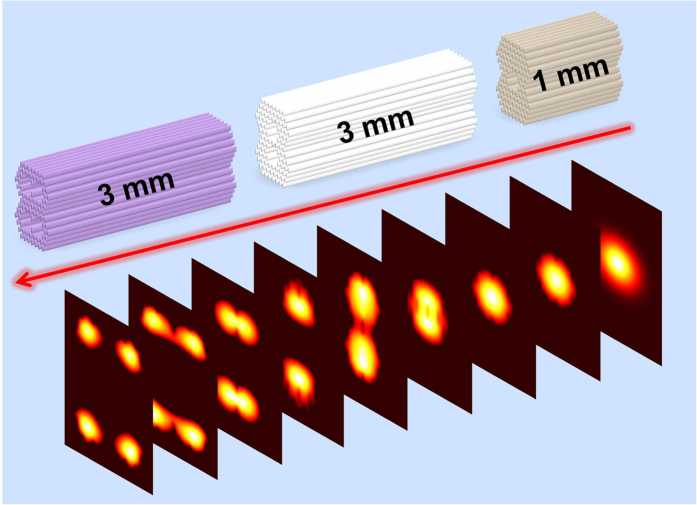
Theoretical modal profile evolution of optical-lattice-like cladding waveguide. Simulated beam profile evolution as the 1064-nm light propagates along the photonic structure in optical-lattice-like cladding waveguide No. 4.

The variation of the refractive index (the contrast between individual modification and the unprocessed crystal) in the tracks is determined to be Δn ~ −0.0035, which results from the combination of the damage induced refractive index reduction and the stress induced refractive index increment in surroundings of the damage tracks. With this value, the spatial distribution of the refractive index can be constituted, as all the parameters are adjusted to maximize agreement between the simulation and the experiment. The theoretical modal profiles of the guiding structure are calculated along both TE and TM polarizations by Rsoft^©^ software based on finite-difference beam propagation method (FD-BPM). As an example, Fig. 4 shows the beam profile evolution of 632.8 nm light propagating in the optical-lattice-like cladding waveguide No. 4 along TM polarization, showing reasonable agreement with the measured modal intensity distributions (Fig. 2(h)).

**Figure 5:**
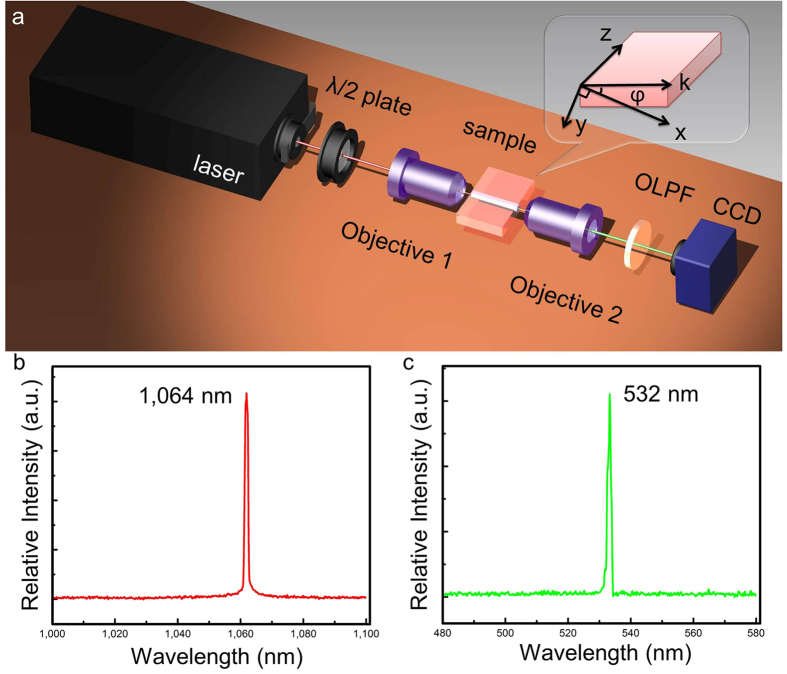
SHG Experiment of optical-lattice-like cladding waveguides in KTP (**a**) Schematic of experimental arrangement for second-harmonic-generation in KTP optical-lattice-like cladding waveguides. The spectra of (**b**) the fundamental laser beam at ~1064 nm and (**c**) the SHG at ~532 nm transmitted through femtosecond-laser inscribed KTP optical-lattice-like cladding waveguides.

In integrated micro-photonic devices, nonlinear optical application is on demand for waveguide structure due to the combination of the original nonlinear properties of substrates and the compact confinement of light propagation within considerably compressed volumes, such as the SHG which is an effective means to generate visible laser under relatively low pump power. In straight channel waveguide, SHG has been realized in many materials. However, it is still limited for monolithic structures capable of both SHG and beam tailoring in nonlinear crystal. In this work, SHG with manipulated guided lasers for the monolithic combination has been realized in cw regime. Figure 5(a) shows the schematic graph of SHG experiment in the KTP optical-lattice-like cladding waveguides through an end-face arrangement pumping by cw laser centering at 1064 nm. The normalized laser spectra of the fundamental (at 1064 nm) and SH (at 532 nm) waves from optical-lattice-like cladding waveguide No. 4 are demonstrated in Fig. 5(b,c), respectively. The generated green laser is TE polarization (i.e. The SH wave is “e”-light.) which is in accordance with the prediction “*e*^ω^ + *o*^ω^ → *e*^2ω^” phase matching configuration from the bulk prototype configuration. It should be noted that for all the structures studied in this work, the laser spectra for fundamental and SH waves are similar to those from the bulk, separately, indicating that the nonlinear properties of the crystal have been well preserved in the optical-lattice-like cladding waveguides.

**Figure 6:**
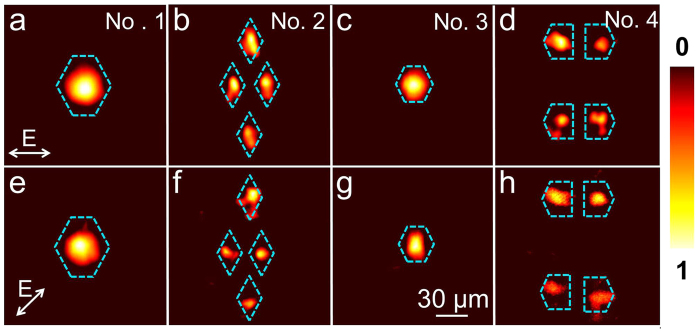
SHG mode of the optical-lattice-like cladding waveguides. Near-field modal profiles of optical-lattice-like cladding waveguides Nos. 1–4 at (**a**–**d**) 532 nm and (**e**,**f**) 1064 nm under 1064 → 532 nm green laser SHG configuration. The inserted arrows are the polarizations.

Figure 6(a–h) show the near-field intensity distribution of the pumping fundamental and output green laser in optical-lattice-like cladding waveguides Nos. 1–4, respectively. The arrows represent the polarization directions of the pumping laser at 1064 nm. As we can see, the frequency doubling has been achieved through the 3D beam splitter. The modal profiles exhibit zero-order modes, which is reasonable since the light filed in lowest-order modes for both fundamental and SH waves have maximum overlap, usually resulting in the highest conversion efficiency.

**Figure 7:**
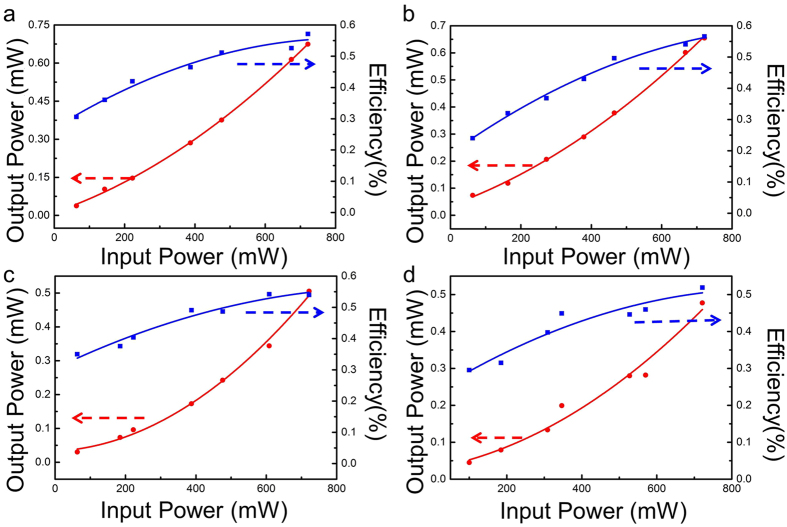
SHG characteristic of the optical-lattice-like cladding waveguides (**a**–**d**) SHG output power and conversion efficiency versus the fundamental pump power of optical-lattice-like cladding waveguides Nos 1–4, respectively.

Figure 7(a–d) depict the generated SH wave powers and the conversion efficiencies at 532 nm as a function of the input fundamental pump power without considering the mismatch coefficient at 1064 nm from the optical-lattice-like cladding waveguides Nos. 1–4, respectively. The solid circles and lines are the experimental data and the linear fit, separately. Taking the mismatch coefficient (i.e. the coupling efficiency) into account, the maximum output power of SH light and corresponding launched pump power at 1064 nm, normalized conversion efficiency for SHG and unconverted absorbed 1064-nm power (i.e. the leaked fundamental laser beam) are listed in Table 1. The maximum green powers have been measured to be 0.675 mW, 0.655 mW, 0.505 mW, 0.477 mW with the absorbed pump power of 9.8 mW, 7.4 mW, 7.1 mW, 5.4 mW for structures Nos. 1–4 corresponding to the normalized conversion efficiency of 11.15%/W, 14.28%/W, 11.30%/W, 13.90%/W, respectively. According to the data of maximum output power of green laser, 1 × 4 beam splitters No. 2 and No. 4 are slightly lower than corresponding straight channel waveguide No. 1 and No. 3 with same incident end-facets, which is reasonable for the additional significant smaller splitter losses. It can be confirmed by the Equation^44^:


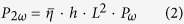


where *P*_*2ω*_ is the SH power at the guiding end, 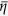 is the normalized conversion efficiency, *h* describes the effect of the waveguide loss and phase-mismatch on the conversion efficiency, *L* is the guiding length of waveguide and *P*_*ω*_ is the incident power coupled into guide. Due to the same phase-mismatch on the conversion efficiency for all structures, *h* is determined by the waveguide loss. As the output power *P*_*2ω*_ is proportional to *h*, we assume that structures possess equal attenuation (i.e. the same *h*). With the higher normalized conversion efficiency, the SH power of beam splitter is higher than corresponding straight channel waveguide, indicating that the nonlinear properties of splitters not merely have been well preserved but also have been enhanced with respect to straight channel waveguide. However, for the data of the normalized conversion efficiency, 1 × 4 beam splitters No. 2 and No. 4 are comparable higher with respect to corresponding straight channel waveguides No. 1 and No. 3, respectively, exhibiting that 1 × 4 beam splitters have superior nonlinear properties to corresponding straight channel waveguides with the same input end-facets. For the straight channel waveguides, the normalized conversion efficiency of waveguide No. 1 is almost the same with the waveguide No. 3, which can be explained by a synergy effect of both the mismatch coefficient and the effective mode index. For an ideal lossless straight channel waveguide, the normalized conversion efficiency can be defined by the Equation^44^:


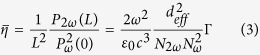


where *ω* denotes the angular frequency of light at the fundamental wavelength, *ε*_*0*_ denotes the permittivity constant and *c* is the speed of the light, *d*_*eff*_ is the effective nonlinear optical coefficient of the material, *N* denotes the effective mode index and *Γ* is the overlap integral of the main field distribution of the fundamental and second-harmonic wave. As the normalized conversion efficiency is proportional to the overlap integral *Γ* and inversely proportional to the effective mode index *N*, it is reasonable to gain the near equal normalized conversion efficiency for both waveguides. In addition, compared with the reported value of the ion-irradiated KTP planar waveguide in cw regime (5.36%/W)^45^, the normalized conversion efficiency is more than twice larger for the optical-lattice-like cladding waveguides. Compared with the previously reported KTP cladding waveguide by fs-laser writing under a pulsed 1064 nm laser pump (0.076%/W/cm^2^)^46^, the normalized conversion efficiency in this work is significantly larger, illustrating that the nonlinear properties has been improved by the design of the structure.

## Discussion

A family of monolithic optical-lattice-like structures consisting of hexagonal or dual-hexagonal lattices of laser-damage bits with reduced refractive index have been designed and fabricated by direct fs-laser writing in a KTP crystal wafer. Periodically arrayed laser-induced tracks serve as depressed cladding structure, which can be used for waveguiding of light. Through such structure, the light propagation can be engineered via the track-confined refractive index profiles, achieving tailored output beam distributions. In this work, 1 × 4 beam splitting capable of not only light tailoring but also efficient SHG has been implemented in a nonlinear KTP crystal, which paves a solid way for a wide variety of applications in many disciplines. On the one hand, from the perspective of fabrication, our work shows the capability of fs-laser micro-fabrication to produce more complex waveguiding devices by integration of more designable elements in a single crystal chip, which would further enlarge the scope of promising applications that can be produced by this fabrication technique. With a certain designs, the optical-lattice-like structure containing a guiding core surrounded by a number of laser-induced tracks with negative index modifications can be used for a wide variety of applications, which could be confirmed by the pattern of structure guidance. This has been realized for the first time in the Nd:YAG laser crystals^40^ and then in the KTP nonlinear crystal studied in our work, which is considerably difficult for crystalline materials (including single crystals and polycrystalline ceramics) since the direct femtosecond laser irradiation typically produces a negative index decrease compared to laser glasses in which fs-laser writing is easily used to produce 3D waveguiding structures. Additionally, it could be extended to other crystal for broader-scope applications of both scientific researches and human life. On the other hand, with Type II phase matching of the fundamental wavelength (@1064 nm) to second harmonic waves (@ 532 nm), the frequency doubling in cw regimes has been achieved through this three-dimensional beam splitter, which demonstrates the nonlinear properties of original KTP crystal have been well preserved within the waveguide volume. It is worth pointing out that 1 × 4 waveguides splitters have superior nonlinear properties compared to the straight channel waveguides with same incident end-facet, which indicates the three-dimensional beam splitter is capable of not only splitting the guidance of the beam but also the improvement of the nonlinear properties. In conclusion, our work demonstrates three-dimensional waveguide splitter in a KTP nonlinear crystal wafer by employing the direct fs-laser writing. Strong capability of fabrication by this simple and flexible technique is also shown in a designable manner for building compact three-dimensional optical-lattice-like structures capable of producing efficient beam divisions. The SHG has been achieved with superior performance depicting that, this splitter structure benefits the frequency doubling. Based on the proposed monolithic optical-lattice-like cladding waveguide, arbitrarily complex 3D beam tailoring devices could be implemented. This work paves a way to produce nonlinear optical devices on chip scales for light guiding, beam splitting and second harmonic generation in dielectric laser crystals for various photonic applications.

## Methods

### Fabrication of optical-lattice-like cladding waveguides

To fabricate the nonlinear waveguide beam splitter in optically polished KTP crystal, which is cut with the dimension of 9 × 7 × 2 mm^3^ to satisfy the Type II (e^ω^ + o^ω^ → e^2ω^) 1064 → 532 nm phase matching (PM) second harmonic generation (SHG) (i.e., θ = 90°, φ = 23.5°) along the 7 mm axis, direct fs-laser writing has been utilized in the sample-scanning approach. An amplified Ti:Sapphire fs-laser (Spitfire, Spectra Physics) which is employed as the laser source delivers linearly-polarized pulses with a temporal duration of 120 fs at a central wavelength of 795 nm and operates at a repetition rate of 1 kHz. The maximum available pulse energy which is controlled by a calibrated neutral density filter placed after a set of half-wave plate and a linear polarizer is 0.17 μJ. The beam is focused by a 40 × microscope objective (N.A. = 0.4) and the laser irradiation is controlled with a mechanical shutter. The sample is located on a computer-controlled 3D-motorized stage that allows scanning the sample at constant velocity of 650 μm/s along 7 mm axis while irradiating from the surface of 9 × 7 mm^2^ with the fs-pulses at certain depth beneath the surface (~70 μm) to form the damage line produced with a transverse length of ~10 μm. The procedure is repeated at different positions of the sample, following certain designed hexagonal and dual-hexagonal photonic structures geometry. The schematic plot of the experimental setup is depicted in Fig. 1a.

### Characterization of Guidance

The guiding properties of the optical-lattice-like cladding waveguides are explored under a typical end-face coupling arrangement. The linear polarized 632.8 nm He-Ne laser, using a half-wave plate to change the polarization, is used as the light source. It is focused by a 20 × microscope object lens and then coupled into one end-face of the structure. Another microscope object lens is utilized as the out-coupler, through which the modal profile at the output of the structure is collected and recorded by a CCD camera. The light powers are measured by a few powermeters. Moreover, the propagation losses of the photonic structures are estimated and determined by directly measurement of the light powers from input and output end-facets taking the Fresnel reflection in the interface of the waveguide end face and the air into account.

### Second Harmonic Generation

The nonlinear performances of the waveguides are characterized by using a similar end-face coupling system compared with the one used for the guiding mode investigation. For the SHG experiments of the structures, a cw linear polarized laser beam at a wavelength of ~1064 nm is used as the pump source. The polarization direction of the fundamental wave is set along the 45° direction in order to mix equal “e” and “o” parts of the fundamental laser for satisfying the 1/2e^ω^ + 1/2o^ω^ for Type II SHG which is controlled by the half-wave plate. A 20 × microscope objective lens with a N.A. of 0.4 is used to couple the beam into one end-face of the structure. The light exiting from the other end-face (i.e. the output of the structure) is aggregated with another 20 × microscope objective lens. After separating from the leaked fundamental laser beam by an optical-low-pass-filter (OLPF) with high transmission of ~90% at 532-nm and reflectivity >99% at 1064 nm, the generated green light signals are detected by the spectrometer with resolution of 0.2 nm, visible CCD camera and the powermeters. The SHG experiment setup with a cw laser source is depicted in Fig. 5a.

## Additional Information

**How to cite this article**: Nie, W. *et al.* Efficient Second Harmonic Generation in 3D Nonlinear Optical-Lattice-Like Cladding Waveguide Splitters by Femtosecond Laser Inscription. *Sci. Rep.* **6**, 22310; doi: 10.1038/srep22310 (2016).

## Figures and Tables

**Table 1:** The nonlinear properties of the optical-lattice-like cladding waveguides.

Number	Launched pump power at 1064 nm (mW)	Leaked pump power at 1064 nm (mW)	Maximum output power at 532 nm (mW)	Normalized conversion efficiency (%/W)
No. 1	613.3	603.5	0.675	11.15
No. 2	613.3	605.9	0.655	14.28
No. 3	635.0	627.9	0.505	11.30
No. 4	635.0	629.6	0.477	13.90
